# Positive Effects of Heme Oxygenase Upregulation on Adiposity and Vascular Dysfunction: Gene Targeting vs. Pharmacologic Therapy

**DOI:** 10.3390/ijms20102514

**Published:** 2019-05-22

**Authors:** Stephen J. Peterson, Rochelle Rubinstein, Mouzam Faroqui, Adnan Raza, Imene Boumaza, Yilun Zhang, David Stec, Nader G. Abraham

**Affiliations:** 1 Department of Medicine, Weill Cornell Medicine, New York, NY 10065, USA; 2 New York Presbyterian Brooklyn Methodist Hospital, Brooklyn, NY 11215, USA; mmf9011@nyp.org (M.F.); asr9034@nyp.org (A.R.); imb9009@nyp.org (I.B.); 3 Departments of Medicine and Pharmacology, New York Medical College, Valhalla, NY 10595, USA; ruchie@verizon.net (R.R.); nader_abraham@nymc.edu (N.G.A.); 4 Tufts University School of Medicine, Boston, MA 02111, USA; Yilun.Zhang@tufts.edu; 5 Department of Physiology and Biophysics at the University of Mississippi Medical Center, Jackson, MI 39216, USA; dstec@umc.edu; 6 Department of Pharmacology, Physiology and Toxicology, Marshall University, Joan Edwards School of Medicine, Huntington, WV 25701, USA

**Keywords:** carbon monoxide, bilirubin, inflammation, cardiovascular disease, diabetes, circulating endothelial progenitor cells, statin, hypertension, cell cycle, gene transfer adipocytes, endothelial cell function, lentiviral transfection, heme oxygenase-1

## Abstract

Objective: Heme oxygenase (HO-1) plays a critical role in adipogenesis and it is important to understand its function in obesity. Many studies have shown that upregulation of HO-1 can affect the biologic parameters in obesity-mediated diabetes, hypertension and vascular endothelial cell function. Thus, we aimed to explore the hypothesis that upregulation of HO-1, using a pharmacologic approach as well as gene targeting, would improve both adiposity and endothelial cell dysfunction by direct targeting of endothelial cells. Our second aim was to compare the short-term effect of a HO-1 inducer, cobalt-protoporphrin IX (CoPP), with the long-term effects of gene targeted therapy on vascular and adipocyte stem cells in obese mice. Method: We examined the effect of CoPP on fat pre-adipocytes and mesenchymal stem cells (MSC) in mice fed a high-fat diet (HFD). We also used a lentiviral construct that expressed heme oxygenase (HO-1) that was under the control of an endothelium specific promoter, vascular endothelium cadherin (VECAD) heme oxygenase (VECAD-HO-1). We targeted endothelial cells using vascular endothelium cadherin/green fluorescent protein fusion construct (VECAD-GFP) as the control. Conditioned media (CM) from endothelial cells (EC) was added to fat derived adipocytes. Additionally, we treated renal interlobar arteries with phenylephrine and dosed cumulative increments of acetylcholine both with and without exposure to CoPP. We did the same vascular reactivity experiments with VECAD-HO-1 lentiviral construct compared to the control. Results: CoPP improved vascular reactivity and decreased adipogenesis compared to the control. MSCs exposed to CM from EC transfected with VECAD-HO-1 showed decreased adipogenesis, smaller lipid droplet size and decreased PPAR-γ, C/EBP and increased Wnt 10b compared to the control. HO-1 upregulation had a direct effect on reducing adipogenesis. This effect was blocked by tin mesoporphrin (SnMP). EC treated with VECAD-HO-1 expressed lower levels of ICAM and VCAM compared to the control, suggesting improved EC function. This also improved ACH induced vascular reactivity. These effects were also reversed by SnMP. The effect of viral transfection was much more specific and sustained than the effects of pharmacologic therapy, CoPP. Conclusion: This study demonstrates that a pharmacological inducer of HO-1 such as CoPP improves endothelial cell function while dampening adipogenesis, but long-term HO-1 expression by direct targeting of endothelial cells by gene transfer therapy may offer a more specific and ideal solution. This was evidenced by smaller healthier adipocytes that had improved insulin sensitivity, suggesting increased adiponectin levels. HO-1 upregulation reestablished the “crosstalk” between perivascular adipose tissue and the vascular system that was lost in the chronic inflammatory state of obesity. This study demonstrates that gene targeting of EC may well be the future direction in treating obesity induced EC dysfunction, with the finding that targeting the vasculature had a direct and sustained effect on adipogenesis.

## 1. Introduction

Obesity remains a crisis of epidemic proportions in this country with a third of our population classified as overweight and another third as obese [1]. Obesity is a chronic inflammatory state with secondary complications of diabetes, metabolic syndrome, hypertension and cardiovascular disease [2]. The mainstay of therapy has long been diet and aerobic exercise, which have not been effective [3]. Pharmacologic therapy has been used to target a 10–12% weight loss goal, but it has also been ineffective [4]. 

HO-1 is the body’s first line of defense against oxidant attack. This cytoprotective enzyme attenuates oxidative stress and plays a critical role in obesity, specifically, in the regulation of adipogenesis. We have shown that obese females with metabolic syndrome and obesity is associated with inflammation that leads to increased levels of reactive oxygen species (ROS). This oxidant attack results in increased levels of oxidized HDL (Ox-HDL) and isoprostane [5]. This was also associated with increases in 20-HETE, TNF α and AngII and decreased adiponectin. Ox-HDL had a more potent effect on adipogenesis than the rest of this biomarker profile. We have also shown that Ox-HDL increases adipogenesis in MSC derived adipocytes, which was further increased with the addition of either 20-HETE or Ang II. This was attenuated by upregulation of HO-1. We have also shown that overexpression of HO-1 can improve fatty liver in obese mice [6]. HO-1 induction has also been shown to improve cardiorenal syndrome in SCID mice [7]. Prior publications have shown that HO-1 upregulation modulates adipogenesis in MSC derived adipocytes [8]. 

CoPP is a potent inducer of HO-1 upregulation, which decreases ROS, increases mitochondrial function and has been shown to reduce body weight [9,10]. It is not specific or long lasting in effect. The same results have been achieved using other pharmacologic inducers such as L4F, an Apo A-1 mimetic protein. CoPP has improved metabolic syndrome and obesity through the same mechanism, with marked reductions in insulin resistance in obese diabetic mice [11,12] (reviewed in [13]) and diabetic rats [14]. We have previously shown that CoPP has additional benefits of improved vascular reactivity [15]. HO-1 also upregulates bilirubin and carbon monoxide, which are anti-inflammatory and anti-apoptotic, respectively [16,17]. This was done with carbon monoxide releasing molecules or by medications that upregulate HO-1 include statins, resveratrol, CoPP and aspirin [10,16,17,18].

While the goal of gene therapy is ultimately to restore energy homeostasis, it is important to understand and identify genes involved with obesity. It is also important to note that more than one gene is involved in obesity and there is currently no “single bullet” to solve this problem. Abraham et al. first described HO-1 as a target gene for diabetes and obesity [19]. Thus, the aim of this study was to compare the short-lived exposure to pharmacologic therapy, determined by half-life, to sustained effects of viral transfection of HO-1. The “crosstalk” between adipose tissue and endothelial cell function is lost in the chronic inflammatory state of obesity. We determined if this is restored by HO-1 upregulation by measuring vascular reactivity [20,21,22]. 

This study seeks to understand the interplay between adiposity and vascular function. Adipose tissue is a very active organ that is capable of secreting adipocytokines (positive and negative) that affect the entire vascular system [20]. Specifically, perivascular adipose tissue has been documented to exert effects on the contractility of the vascular beds that they surround [21,22]. In normal circumstances, this perivascular adipose tissue can help modulate vascular tone. Conversely, the endothelium has a key role in modulating adipose tissue expansion [23,24]. In morbid obesity, this crosstalk appears to be dysfunctional with the consequent release of inflammatory adipokines as we showed in a murine model of obesity [25]. 

The goal of this report is to compare the effects of short-term therapy, CoPP, vs. the long-term effects of gene transfer therapy for HO-1 upregulation on vascular reactivity. We used a lentiviral construct under the control of an endothelium specific promoter, vascular endothelium-cadherin heme oxygease (VECAD-HO-1). VECAD-GFP was used as the control. Additionally, the present study is to test chemical inducers of HO-1 such as CoPP vs. gene targeting of EC and it includes parameters like vascular reactivity and adipokine levels and their effect on adipogenesis. We also evaluated the mechanism by testing CM from EC transfected by HO-1-VECAD and its effect on adipogenesis. 

## 2. Results

### 2.1. Effect of Obesity on Adipogenesis

**Figure 1:**
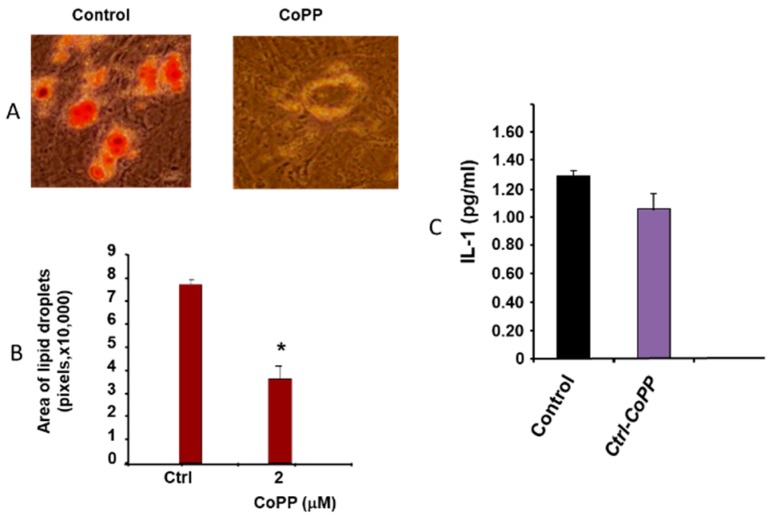
Effect of HO-1-induction by CoPP on adipogenic differentiation of bone-marrow-MSC-derived adipogenesis * (*p* < 0.05). This shows the effect of CoPP on the adipogenic differentiation of MSC derived from mice fed an HFD. (**A**,**B**) show the effect of CoPP on the numbers of adipocytes as well as the size of the adipocytes. (**C**) shows the decrease in IL-6.

Figure 1 shows the effect of obesity in mice fed an HFD on MSC-derived adipocytes (Figure 1A). Figure 1A,B show the effect of CoPP on both the number of adipocytes as well as the effect on lipid size * (*p* < 0.05). CoPP administration decreases both the number and size of adipocytes. Figure 1C shows the decrease in IL-1 compared to the control. This suggests that CoPP mediated HO-1 changes to the phenotype to naive healthy adipocytes, i.e., smaller adipocytes with lower levels of IL-1.

### 2.2. Vascular Reactivity

**Figure 2:**
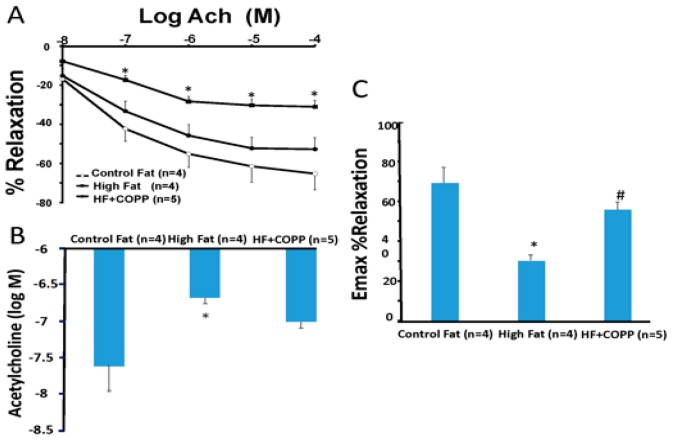
(**A**) shows the dose-response curves for acetylcholine-induced vasorelaxation of renal artery rings precontracted with phenylephrine. In mice with high fat diets, the acetylcholine dose-response curve was shifted down, and the 50% effective concentration (**B**) increased from 10^−7^ M to 10^−4^ M in rings isolated from control mice. Treatment of mice with CoPP, a HO-1 inducer, prevented high fat-induced impairment of endothelial function as the dose-response curves (**C**) * (*p* < 0.05 vs. lean and VECAD-HO-1); # (*p* < 0.05 vs. high fat).

Figure 2A shows the dose-response curves for acetylcholine-induced vasorelaxation of renal artery rings precontracted with phenylephrine. In mice with high fat diet, the acetylcholine dose-response curve was shifted to the right, and the 50% effective concentration (Figure 2B) increased from 10^−7^ M to 10^−4^ M in rings isolated from control mice. Treatment of mice with CoPP, a HO-1 inducer, prevented high fat-induced impairment of endothelial function as the dose-response curves and the 50% effective concentration for acetylcholine were not different from those of control mice. Furthermore, the impairment of endothelial-dependent vasorelaxation in isolated renal artery rings of high fat mice would be prevented in VECAD-HO-1 mice (Figure 2A,B,C). The dose response curves were also shifted to the right compared to high fat control.

### 2.3. The Effect of HO-1 Lenti-Virus on Gene Expression

**Figure 3:**
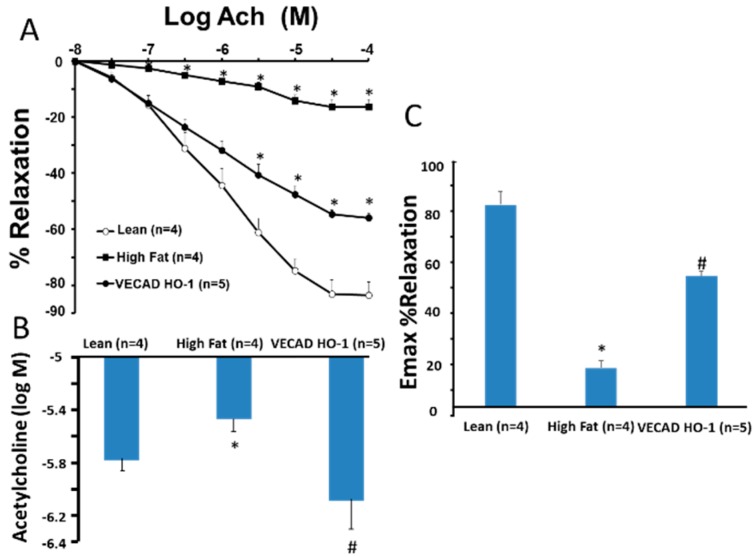
Effect of lentiviral-VECAD-HO-1 on vascular reactivity. Shows the impairment of endothelial-dependent vasorelaxation isolated from lean mice, obese mice, and obese treated with VECAD-HO-1 in response to ACh in isolated renal artery rings of GFP mice. This was corrected in VECAD-HO-1 mice (**A**–**C**). * (*p* < 0.05 vs. lean and VECAD-HO-1); # (*p* < 0.05 vs. high fat).

In order to identify the effect of HO-1 gene targeting on vascular EC, we examined the relationship between EC function of EC transfected (VECAD-HO-1) with adipocyte health and compared this to the control (VECAD-GFP). Western blot analysis (Figure 3A) shows protein expression levels following treatment with VECAD-HO-1. 

### 2.4. Lenti-VECAD-HO-1A Transduction Increase HO-1, Decreases Adhesion Molecules

**Figure 4:**
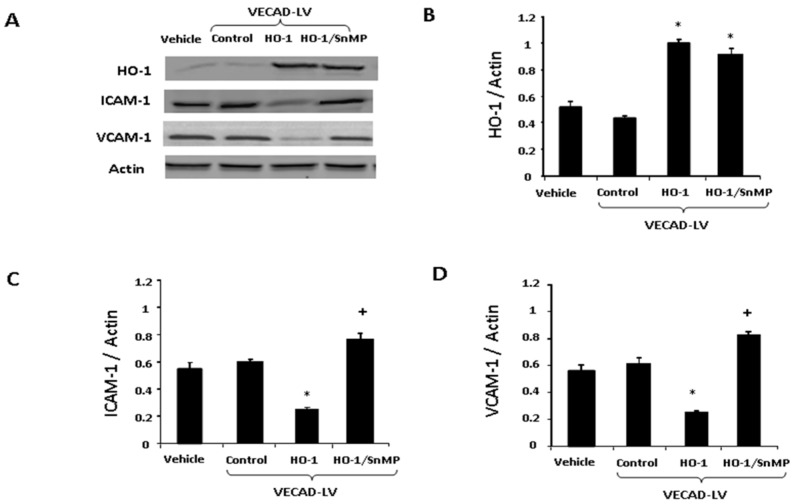
(**A**) Western blot analysis of HO-1, ICAM-1, and VCAM-1 expression following treatment of EC with VECAD-GFP (Control), VECAD-HO-1, and VECAD-HO-1 + SnMP. (**B**–**D**) Graphs depicting HO-1, ICAM-1, and VCAM-1 expression following treatment of EC with VECAD-GFP (Control), VECAD-HO-1, and VECAD-HO-1 + SnMP (* vs. Control *p* < 0.05, + vs. HO-1 *p* < 0.05).

Lenti-VECAD-HO-1 transfection increased expression of HO-1 (Figure 4B) and decreased expression of both ICAM-1 (Figure 4C) and VCAM-1 (Figure 4D) when compared to the control. Addition of SnMP elevated (*p* < 0.05) ICAM-1 and VCAM-1 when compared to the control (Figure 4).

### 2.5. Effect of CM from EC on Adipogenesis in MSCs

**Figure 5:**
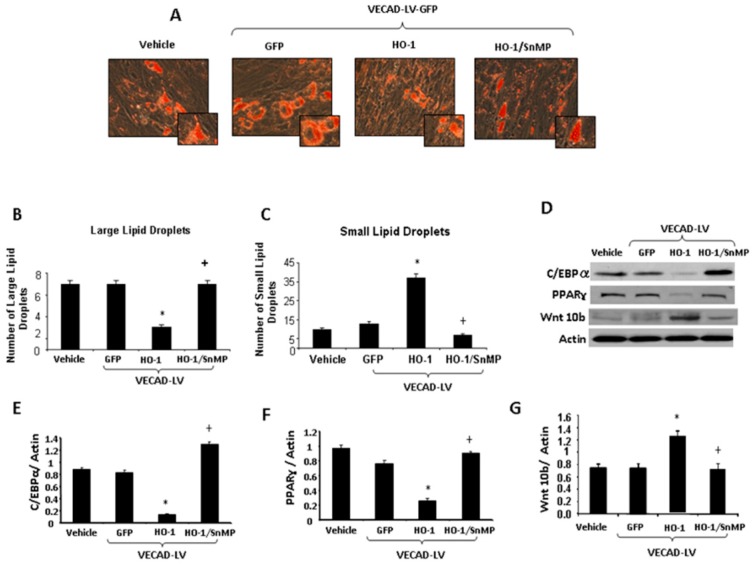
(**A**) Adipogenesis in MSC-derived adipocytes following administration of CM from EC treated with VECAD-GFP, VECAD-HO-1, and VECAD-HO-1 + SnMP. (**B**,**C**) Lipid droplet size of MSC-derived adipocytes following administration of CM from EC treated with VECAD-GFP, VECAD-HO-1, and VECAD-HO-1 + SnMP. (**D**) Western blot bands of C/EBP-α, PPAR-γ and Wnt 10b expression from MSC-derived adipocytes following administration of CM from EC treated with VECAD-GFP, VECAD-HO-1, and VECAD-HO-1 + SnMP. (**E**–**G**) Graphs depicting C/EBP-α, PPAR-γ and Wnt 10b expression from MSC-derived adipocytes following administration of CM from EC treated with VECAD-GFP, VECAD-HO-1, and VECAD-HO-1 + SnMP (*, vs. GFP *p* < 0.05, +, vs. HO-1 *p* < 0.05).

Figure 5A illustrates that adipogenesis is reduced in MSCs exposed to CM from VECAD-HO-1 EC. The reduction was reversed by SnMP. To confirm that increased levels of VECAD-HO-1 resulted in decreased adipogenesis, we stained lipid droplets with BODIPY/DAPI (Figure 5B,C). VECAD-HO-1 treatment decreased (*p* < 0.05) the number of very large lipid droplets, and increased the number of small lipid droplets when compared with the vehicle (Figure 5B,C). In contrast, SnMP significantly (*p* < 0.05) increased the number of large lipid droplets and decreased the number of small lipid droplets compared to VECAD-HO-1, indicating that this effect is mediated by increased HO activity. Figure 5D show the expression levels of the downstream adipocyte differentiation markers PPAR-γ and C/EBP-α and negative regulator of adipogenesis, Wnt 10b in MSC-derived adipocytes. The expression of C/EBP-α and PPAR-γ is reduced by VECAD-HO-1 CM and increased by VECAD-HO-1/SnMP CM (Figure 5D–F). Conversely, Wnt 10b expression is increased by VECAD-HO-1 CM and decreased by VECAD-HO-1/SnMP (Figure 5G).

### 2.6. Analysis of Biomarkers from CM of EC

**Figure 6:**
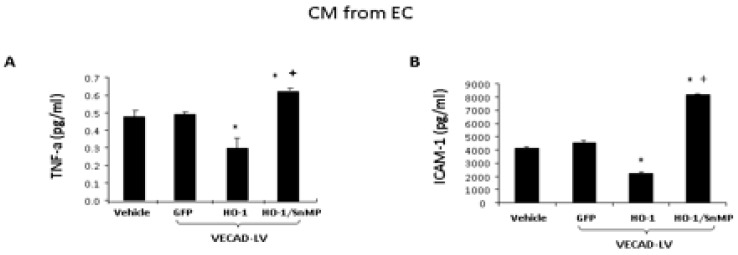
(**A**,**B**) Cytokine array analysis of TNF-α and ICAM-1 following treatment of HMEC-1 with VECAD-GFP, VECAD-HO-1, and VECAD-HO-1 + SnMP (* vs. GFP *p* < 0.05, + vs. HO-1 *p* < 0.05).

As seen in Figure 6, we examined the levels of the inflammatory cytokine (A)-TNF-α, (B)-adhesion molecule ICAM-1, CM from cells treated with VECAD-HO-1 resulted in a decrease in TNF-α and ICAM-1 when compared to the control (Figure 6). In contrast, CM from cells treated with VECAD-HO-1 and SnMP resulted in an increase in TNF-α and ICAM-1 levels compared to the control (*p* < 0.05).

## 3. Discussion

This is the first study to compare repeated administration of a pharmacological inducer of HO-1, such as CoPP, to long term HO-1 expression by direct targeting of endothelial cells in mice fed a HFD with a lentiviral HO-1 construct. Both the HO-1 inducer, CoPP and the lentiviral HO-1 construct led to decreased adipocyte-derived hypertrophy as measured by size of lipid droplets. and improved vascular dysfunction of obese mice. It also improved metabolic parameters and attenuated levels of inflammatory cytokines. CoPP decreased the size of lipid droplets and the level of IL-1 while improving acetylcholine-mediated vascular relaxation. The transfection of VECAD-HO-1 further increased the numbers of adipocytes of small cell size as measured by lipid droplet size (*p* < 0.05), decreased adipokines, TNF, ICAM, VCAM and improved vascular relaxation to acetylcholine similar to HF mice administered CoPP in a more specific and sustained fashion (Figure 2, Figure 4 and Figure 5).

Gene transfer involves a process of inserting specific genes into cells. Gene transfer has been used for years in oncology [26,27] and only recently appreciated as a treatment of obesity [28,29]. Gene transfer is used to address cells that either under express a gene or may be totally deficient in that gene. Upregulation of HO-1 results in heme degradation to iron, bilirubin (an anti-oxidant), and carbon monoxide (anti-inflammatory and anti-apoptotic properties) [10,13]. There are two requirements for HO-1 successful gene transfer. The first is safe transfer via vector, in this case we used a lentiviral construct. The second consideration is site specific delivery. Here the site was viral transfection into endothelial cells. Lentiviruses are from the HIV viral line. This retrovirus can infect cells at multiple stages in the cell cycle and has multiple receptor sites on cells for delivery. It is encoded and integrated into the cell to ensure long term expression of the gene. Most pharmacologic therapies used for obesity are oral medications that require daily administration. Gene transfer therapy would offer long term gene expression [30]. The VECAD promoter is a specific promoter to deliver HO-1 and is exclusively expressed in endothelial cells. This study shows that direct targeting of endothelial cells down regulated adipogenesis with the creation of smaller, more insulin sensitive adipocytes. Gene targeting by VECAD-HO-1 reduced ICAM-1, VCAM-1, (Figure 3), and ICAM and TNFα levels culture media from endothelial cells (Figure 5). This sustained gene overexpression resulted in healthier adipocytes with a resultant decrease in inflammatory adipokines (Figure 5). VECAD-GFP was used as the control. Increased HO-1 levels were reduced by SnMP. This SnMP reversal shows that HO levels and HO-1 activity are responsible for this effect. 

The size of the adipocyte is an indication of function. Smaller adipocytes are insulin sensitive and release higher amounts of adiponectin [31,32]. Larger “necrotic” adipocytes are insulin resistant and release markedly less adiponectin than their healthy counterparts [33]. Adiponectin is an adipokine responsible for improved insulin sensitivity [34,35,36]. Adipocyte size is also determined by C/EBP-α and PPAR-γ, which is a downstream activator of inflammatory cytokines like MCP-1, which results in larger more necrotic adipocytes [37,38,39,40]. Conversely, Wnt 10b decreases adipocyte differentiation and down regulates PPAR-γ, inhibiting the process of adipogenesis. VECAD-HO-1 targeted EC control media (CM) decreased C/EBP-α and PPAR-γ, while increasing Wnt 10b. This effect was negated by VECAD-HO-1/SnMP.

This study underscores the interplay between adiposity and vascular function. Adipose tissue is a very active organ that is capable of secreting adipokines (positive and negative) that affect the entire vascular system [20]. Specifically, perivascular adipose tissue has been documented to exert effects on the contractility of the vascular beds that they surround [21,22]. In normal circumstances this perivascular adipose tissue can help modulate vascular tone. Conversely, the endothelium has a key role in modulating adipose tissue expansion [23,24]. In morbid obesity, this crosstalk appears to be dysfunctional with the consequent release of inflammatory adipokines, as we showed in this murine model of obesity [25]. Much was learned about angiogenesis with the discovery of vascular endothelial growth factor (VEGF). VEGF appears to be intimately involved with wound repair [41] and wound repair requires angiogenesis [42,43]. Others have shown that healthy ECs produce VEGF, which helps control body weight. It has been described as a paracrine effect between the vasculature and adipose tissue. 

Adipose tissue expansion requires recruitment of new vasculature, namely, capillaries. VEGF has also been shown to decrease expression of VCAM-1 [44,45]. We saw elevated levels of ICAM, VCAM, TNF, C/EBP, PPAR gamma and low Wnt 10b and adiponectin. This was accompanied by large necrotic unhealthy adipocytes. This effect was blunted by CoPP upregulation of HO-1 and blocked by SnMP, showing that the effect was HO-1 activity and upregulation. In addition, we showed that ACH-mediated vascular relaxation was improved by HO-1 upregulation. Viral transfection of HO-1 was even more dramatic in the reduction of inflammatory adipokines, and had an even greater response on ACH-mediated vascular relaxation in a much more sustained fashion. In effect, HO-1 upregulation reduced inflammatory adipokines and improved function that resulted in improved vascular relaxation. While many studies underscore the interplay between perivascular fat and vascular function, we believe this is the first study to document that HO-1 upregulation can restore this “crosstalk” and re-establish normalcy. We have shown that viral transfection of EC by HO-1 lowered ICAM, VCAM and inflammatory adipokines that reduced adipogenesis and limited adipose tissue expansion. This study also shows the importance of gene therapy as a new direction in the intervention of the chronic inflammatory state of obesity and its consequent vascular dysfunction.

**Figure 7:**
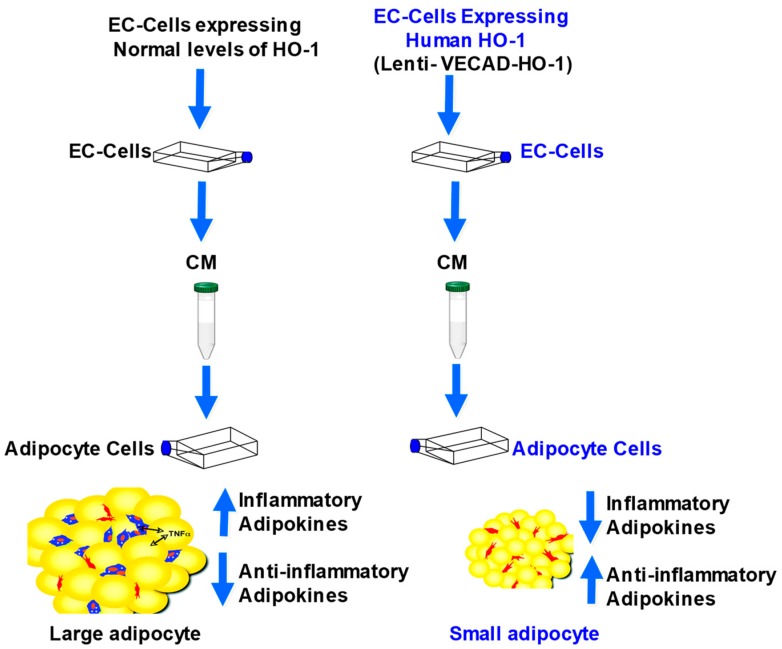
Scheme illustrating the sustained effect of VECAD-HO-1, CM from EC-transfected with HO-1 release substances that increase healthy adipocytes, while CM from control EC add to adipocyte culture, increases adipogenesis and large unhealthy adipocytes.

In summary, the direct targeting of EC by HO-1 reduces inflammatory adipocytokines, while also improving vascular function. Direct targeting reduced adipogenesis in a much more sustained and specific way (Figure 7). HO-1 upregulation re-established the “crosstalk” between perivascular adipose tissue and the vascular system, evidenced by reduced adipogenesis and improved vascular relaxation. Targeting ECs had a direct and sustained effect on reducing adipogenesis. This study demonstrates that gene targeting of EC may well be the future direction in treating obesity induced EC dysfunction, with the conclusion that targeting the vasculature had a direct effect on adipogenesis in a more specific and sustained fashion than pharmacologic intervention. 

## 4. Material and Methods

### 4.1. Cell Culture and Treatment and Animal Studies

EC were purchased from ATCC (Manassas, VA, USA) and maintained at 37 °C and 5% CO_2_ in vascular basal medium containing 1% penicillin/streptomycin and microvascular growth supplement. Adipogenesis was then induced in MSCs in 10% conditioned media (CM) from EC.

In order to specifically target the endothelium with HO-1, EC were treated for three days with lentiviral vectors. The lentiviral vectors were constructed using the LentiMax system (Lentigen, Baltimore, MD, USA) and expressed green fluorescent protein (GFP) with or without human HO-1 under the control of the endothelial-specific promoter VECAD [46]. Cells were infected with the lentiviral vector (2 μL of 10^9^ transducing units [TU]/mL) carrying either the HO-1 construct with GFP under the control of the endothelium-specific promoter VECAD (VECAD-HO-1) (Lentigen). The CM from EC was collected and stored at −80 °C until analyzed. Fat derived primary adipocytes were isolated as described in [47]. Fat primary adipocytes were grown until 80% confluent in a T-75 flask and the medium was replaced with adipogenic medium. The adipogenic media consisted of complete culture medium supplemented with DMEM-high glucose, 10% (*v*/*v*) FBS, 10 μg/mL insulin, 0.5 mM dexamethasone (Sigma–Aldrich, St. Louis, MO, USA) and 0.1 mM indomethacin (Sigma–Aldrich, St. Louis, MO, USA). Adipocytes and MSC-derived adipocytes were cultured in adipogenic differentiation media, treated with 10% CM from the EC. Media was changed every 2 days for 14 days. The CM from MSC-derived adipocytes was collected and stored at −80 °C until analyzed.

### 4.2. Oil Red O Staining and Lipid Droplet Size

For Oil Red O staining, 0.21% Oil Red O in 100% isopropanol (Sigma–Aldrich, St. Louis, MO, USA) was used. After 14 days, fat-derived adipocytes were briefly fixed in 10% formaldehyde, washed in Oil-red O for 10 min, and rinsed with 60% isopropanol (Sigma–Aldrich, St. Louis, MO, USA). The Oil Red O was eluted by adding 100% isopropanol for 10 min and OD was measured at 490 nm. After induction of adipogenesis, lipid droplets were stained with 2μM BODIPY 493/503 (Molecular Probes, Eugene, OR, USA). Cell size was measured using an ImagePro Analyzer (MediaCybernetics, Inc., Milan, Italy). The classification of the size of lipid droplets was based on size by area (pixels).

### 4.3. Animal Treatment with Lentiviral-VECAD-HO-1

Six-week old C57 male mice were fed a HFD for 22 weeks, with manifestation of fatty liver, i.e., NASH was expressed. Mice were divided into 6 groups of 4 animals each: (1) control mice on normal chow diets; (2) HFD; (3) HFD treated for 8 weeks with lentiviral vector expressed human HO-1 under the control of endothelial-specific promoter VECAD injected into the left renal artery (100 μL of 10^12^ pfu/mL of 100 g mice under anesthesia); (4) HFD treated with cobalt protoporphyrin (CoPP) for the last 8 weeks at a dose of 5 mg/100g bw, once a week (subcutaneously). At the end of the study, the mice were euthanized and renal interlobar arteries were collected for further evaluation after contraction with phenylephrine and relaxation with ACh. All animal experiments followed the New York Medical College, IACUC Institutional approved protocol (August 2015) in accordance with the National Institutes of Health guidelines.

### 4.4. Assessment of Agonist-Induced Vasorelaxation in Renal Interlobar Arteries Rings

Renal interlobar arteries were cut into ring segments (2 mm in length) and mounted on 40-µm stainless steel wires in the chambers of a myograph (J.P. Trading) for measurement of isometric tension. The rings were bathed in Krebs buffer (37 °C) containing indomethacin (1 μmol/L) and gassed with 95% O_2_-5% CO_2_, unless indicated otherwise. After a 30-min equilibration interval, the rings were set to an internal circumference equivalent to 90% of the relaxed circumference under a transmural pressure of 100 mmHg and were allowed to stabilize for 20–30 min. The rings were then depolarized with KCl (60 mM) to evaluate maximal contraction. After the vascular preparations were washed with Krebs buffer, they were contracted with phenylephrine (10^−6^ M). When the contractile response was stabilized (steady-state phase, 12–15 min), vasorelaxation responses to cumulative increments in the ACh (10^−9^–10^−4^ mol/L) concentrations were examined in the oxygenated Krebs buffer.

### 4.5. Western Blot Analysis

Cells were harvested and homogenated using a pH 7.5 cell lysis buffer consisting of 10 mM phosphate buffer, 250 mM sucrose, 1 mM EDTA, 0.1 mM phenylmethylsulfonylfluoride, and 0.1% (*v*/*v*) tergitol. Homogenates were centrifuged at 27,000 *g* for 10 min and the supernatant was decanted. The concentrations of proteins from total cell lysate were quantified using the Bradford Method prior to immunoblotting analysis. 20 μg of proteins were separated by 12% SDS-PAGE and transferred to a nitrocellulose membrane. Immunoblotting was performed as previously described [48]. The lysate was used to measure the protein levels of HO-1, ICAM-1, VCAM-1, C/EBP α, Wnt 10b and PPAR-γ. b-Actin was used to ensure adequate sample loading for all Western blots.

### 4.6. Cytokines Array

Tumor necrosis factor-a (TNF-α,), was determined using enzyme-linked immunosorbent assays (Assay Gate, Ijamsville, MD, USA) as previously described [49].

### 4.7. Statistical Analyses

Data are expressed as means ± S.E.M. Significance of difference in mean values was determined using one-way analysis of variance followed by the Newman-Keul’s post hoc test. *p* < 0.05 was considered to be significant.
